# An Overview of the Thrips Fauna of the “Góra Bucze” Landscape-Nature Complex in Western Carpathians (Poland)

**DOI:** 10.3390/insects15110881

**Published:** 2024-11-09

**Authors:** Marta Olczyk, Halina Kucharczyk, Maria Pobożniak

**Affiliations:** 1 Department of Plant Protection, Faculty of Biotechnology and Horticulture, University of Agriculture, al. 29 Listopada 54, 31-425 Cracow, Poland; marta.olczyk@urk.edu.pl; 2 Department of Zoology and Nature Protection, Faculty of Biology and Biotechnology, Maria Curie-Skłodowska University, ul. Akademicka 19, 20-033 Lublin, Poland; halina.kucharczyk@mail.umcs.pl

**Keywords:** species richness, diversity, evenness, meadows, Terebrantia, graminicolous, species

## Abstract

The degree of knowledge of Thysanoptera in the various ranges of the Carpathian Mountains is uneven. The aim of this research was to identify the Thysanoptera fauna in the Góra (Mount) Bucze natural-landscape complex. Three meadow–pasture complexes were selected for the study. Various indicators were used to assess the evenness of the thrips assemblages. A total of 30 thrips species from Aeolothripidae, Thripidae, and Phaeolothripidae were collected, including 26 herbivorous taxa and 4 zoophages. Among herbivores, 11 species were associated with monocotyledonous plants, 12 chose flowers of dicotyledonous herbaceous plants, and 3 preferred leaves, including 2 species which were dendrophilous. The most abundant grass-living species among them were *Chirothrips manicatus*, *Aptinothrips rufus*, *Chirothrips hamatus,* and *Anaphothrips obscurus*. Flower-living species collected from all sites included *Frankliniella intonsa*, *Neohydatothrips gracillicornis*, and species of the genus *Thrips*: *T. fuscipennis*, *T. major*, and *T. physapus*. All reported species, except *Limothrips cerealium*, were found in various mountain ranges of the Polish Carpathians. *Neohydatothrips abnormis*, considered rare and found only at the Babia Góra massif, was caught in site 2. The values of the biodiversity indices are indicated for the three study sites.

## Simple Summary

Within the Carpathian Mountains in Poland, 125 species of thrips have been identified so far, while 211 species are known from all parts of this mountain area in Europe. Fauna of only a few ranges may be regarded as well known: the Mały Beskids Hills, the Sądecki Beskids Hills, the Niski Beskids Hills, and the Babia Góra massif. In 2014–2015, we conducted a study of the thrips fauna in the Góra (Mount) Bucze natural and landscape complex, which is the northernmost area of the Western Outer Carpathian arc. Three meadow–pasture complexes of mixed use were selected for the study. A total of 30 thrips species from Aeolothripidae, Thripidae, and Phaeolothripidae were collected, including 26 herbivorous taxa and 4 zoophages. The most abundant grassland species among them were *Chirothrips manicatus*, *Aptinothrips rufus*, *Chirothrips hamatus,* and *Anaphothrips obscurus*. The flower-living species collected from all sites included *Frankliniella intonsa*, *Neohydatothrips gracillicornis*, and species of the *Thrips* genus: *T. fuscipennis*, *T. major*, and *T. physapus*. All species identified by us, except *Limothrips cerealium*, were found in various mountain ranges of the Polish Carpathians. In turn, *Neohydatothrips abnormis*, considered a rare species, has so far been found within the Carpathians only on the Babia Góra massif.

## 1. Introduction

Although thrips are commonly viewed primarily as agricultural pests [1,2], their role in ecosystems is much broader [3]. They are a diverse group of insects in terms of habitat and food preferences and participate in many ecological interactions [4]. They function as pollinators [5], predators [6,7], facultative ectoparasites [8], commensals living near birds and mammals [9,10], and mycetophagous [1]. Only less than 1% of thrips populations are thought to cause economic losses through direct feeding or disease transmission [3,11,12].

Thysanoptera is part of all biocoenoses, from the first stages of succession to the climax [13]. Thrips communities reflect their environment’s condition and ecological stability, and some species can be used as bioindicators of changes occurring in them [13,14]. The most anthropogenic research sites are characterized by the lowest values of species richness, species diversity, and evenness. In contrast, meadow communities and those with intact forest stands are characterized by higher species richness. This is most likely because meadows are inhabited by more plant species than forests [13].

Approximately 6300 species of thrips are currently recognized worldwide, which are included in two suborders: Terebrantia and Tubulifera. The latter comprises 65% of all thrips species and is placed in a single family called Phlaeothripidae. Mycophagous taxa, feeding on hyphae and fungal spores, comprise a significant proportion of Tubulifera. This also includes one of the most abundant in the genus Haplothrips, represented both by dendrophilous species found exclusively on trees and those feeding on herbaceous plants. The suborder Terebrantia includes eight extant families, of which three occur in Poland: Aeolothripidae, Melanthripidae, and Thripidae. The first of these is a concentration of predatory species; the second, which is the least numerous, includes three flower-living species; while the last and most numerous contains mainly species associated with herbaceous plants, as well as dendrophilous species that feed on the leaves and flowers of trees [3,15,16].

Thus far, 226 species of thrips have been found in Poland [17]. Although thrips are usually quite abundant in plant communities, they are mostly overlooked in faunal and ecological studies due to their small size (1–3 mm) and their oft-hidden lifestyle. Knowledge of both their biology and distribution is insufficient, and the degree of knowledge about thrips in individual regions of Poland is poor and uneven. Thanks to the studies of some authors [18,19,20,21,22,23,24,25,26,27,28,29,30], the fauna of Thysanoptera of central, eastern, and southeastern Poland is best known in this respect. Less-explored areas include mountain and foothill areas, especially in the southwestern part of the country. Mountain ecosystems are characterized by a high level of species diversity and may be the habitat of some unique insect species [31].

From the Carpathians in Poland, 125 species of thrips have been recorded so far [32], while 211 species are known from all parts of this mountain range in Europe [32,33,34]. From the Polish Carpathians, data come from the Mały Beskids and Silesian Beskids [35,36], the Polish part of the Babia Góra massif, the Tatras, the Pieniny Mountains [28,37], the Sądecki Beskids [38,39,40], the Niski Beskids, the Bieszczady Mountains, and the Sanocko-Turczańskie Mountains [41,42]. As emphasized by Kucharczyk and Stanisławek [32], systematic studies were conducted only in the Mały Beskids mountain range, the Babia Góra massif, the Niski Beskid mountain range, and the Sanocko-Turczańskie Mountains in the vicinity of Lesko. In the other Carpathian ranges, research has been carried out irregularly or not at all. The thrips fauna of the Sudetes (a mountain chain in western and southern Poland, northern Bohemia, and with a small patch located in Germany) are also poorly known. In the Western Sudetes, Zawirska [19] listed eight species. Additional data on 38 taxa from the Central and Eastern Sudetes were provided by Stanislawek and Kucharczyk [43]. In total, together with data from the neighboring range of the Rudawy (German: Landeshuter Kamm) in Germany, 73 species have been reported [44].

Considering the insufficient knowledge of the Carpathian thrips fauna in Poland, our research aimed to determine species richness and dominance structure and to perform a chronological and ecological analysis of the collected material.

## 2. Materials and Methods

### 2.1. Study Area

#### 2.1.1. Natural Landscape Complex

The research was conducted in the area of the natural landscape complex of Góra (Mount) Bucze (417.8 m above sea level), which rises on the right bank of the Brennica River in the village of Górki Wielkie, in the northern part of the Brenna commune (49°46′ N, 18°50′ E). This hill is located on the border of the Beskids and Silesian foothills and is the northernmost area of the Western Outer Carpathian arc.

Some of the most entomologically interesting areas are those where different natural habitats occur side by side. This certainly includes Góra Bucze, where the dominant plant community is the subcontinental oak–hornbeam forest (*Tilio-Carpinetum betuli*). It is formed by a multispecies, fertile forest with a predominance of lime trees in the stand and a rich undergrowth. On the northeastern slope of the hill there is a fragment of spruce forest, while on the southern edges of the hill, between the forest and the hay meadows (*Arrhenatheretum elatioris*), scrubland with sun-living plants is found. On northern, western, southwestern, and eastern slopes, the ryegrass meadow of *Arrhenatheretum elatioris* is common [45]. Due to its location in the foothills, Góra Bucze is classified as a moderately warm climate zone, with average annual temperatures ranging from 6 to 8 °C [46]. The climate of this area is milder than that of the neighboring mountains. A characteristic feature of the phytocoenoses of Góra Bucze is the mosaic of forest, scrub, and meadow communities [47]. To date, 16 plant complexes have been distinguished in it [48], in which 449 plant species have been found, including 36 plant species under species protection, 63 species that are rare or endangered on a regional or national scale, and 8 species (mainly from the orchid family) that are on the European Red List of Plants [49,50]. Most of the rare and endangered plants are species of fertile deciduous forests; some are plants associated with humid or sward habitats.

**Figure 1:**
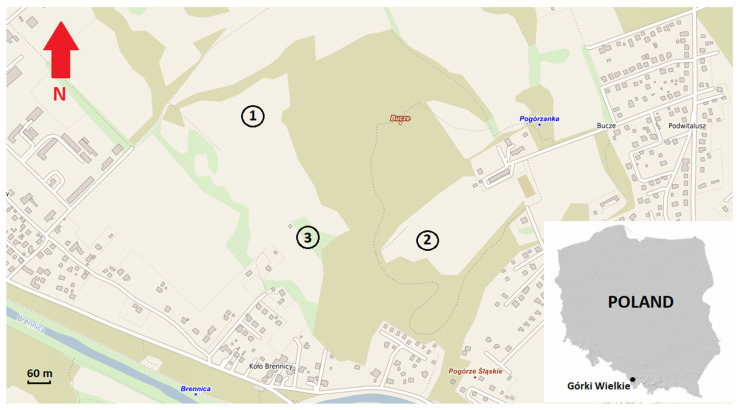
Location of the study sites, Góra Bucze, Poland.

A pastoral economy was developed in the region and in the area of Góra Bucze as early as the end of the 15th century and persisted for a very long time [51]. Nowadays, sheep still graze in some areas and others are seasonally mown. In turn, in those places where grazing has been forbidden, a succession of other species, mainly in meadows and on the border with forests and woodland, takes place. Three meadow–pasture communities adjacent to nearby oak–hornbeam forests were selected for the study of thrips assemblages occurring on Góra Bucze (Figure 1).

#### 2.1.2. Study Sites

Study site 1 was located on the mountain’s western slope and was an anthropogenic habitat used for mowing and occasionally as pasture. It contained meadow species characteristic of the alliance *Arrhenatherion elatioris* and patches of the fox sedge complex *Carex vulpina* [48]. The most abundant were tufted vetch *Vicia cracca*, smooth bedstraw *Galium mollugo*, red clover *Trifolium pratense*, meadow buttercup *Ranunculus acris*, common hornwort *Cerastium holosteoides*, common tansy *Tanacetum vulgare*, common dandelion *Taraxacum officinale*, spreading bellflower *Campanula patula*, St. John’s wort *Hypericum perforatum*, ragged robin *Lychnis floscuculi*, and wild chamomile *Chamomilla recutita*.

Study site 2 was a fresh ryegrass meadow of the alliance *Arrhenatherion elatioris*. It overgrew the western and southern slopes of the hill, below the forest line, occupying habitats that were not very humid or fertile. The floristic composition of this seminatural phytocoenosis was formed under the influence of moderate trampling and as a result of cyclic mowing (twice a year or more often). The ryegrass meadow undergrowth was dominated by various grass species, such as false oat-grass *Arrhenatherum elatius*, cocksfoot *Dactylis glomerata*, and meadow foxtail *Alopecurus pratensis*. They were accompanied by, among others, tufted vetch *V. cracca*, smooth bedstraw *G. mollugo*, and red clover *T. pratense* [48].

Study site 3 was located on the western slope of Góra Bucze, in an area previously used for mowing, pasture, or fallow land. It featured the presence of plant communities belonging to the alliance of *Arrhenatherion elatioris* such as a large patch of wild elderberry *Sambucus nigra*, which overgrew the humid edges of the forest, close to a water exudation. Small clumps of wood club-rush *Scirpus sylvaticus* were also present. Regular grazing by cattle and sheep resulted in the overfertilization of the area and the presence of ruderal plants such as stinging nettle *Urtica dioica* or the herb Robert *Geranium robertianum* and wood small-reed *Calamagrostietum epigeji* [48].

### 2.2. Data Collection

The research was conducted in 2014 and 2015. Thrips were collected on eight dates at approximately fortnightly intervals from the first day of May to the end of August on sunny and warm days between 10 a.m. and 3 p.m. In a temperate climate, this time of day allows for a maximum collection of thrips individuals [52].

Four randomly selected transects, each 100 m long and approximately 3 m wide, were set at each of the three study sites. Thrips were caught using a standard sweeping net (30 cm diameter, 91 cm handle, and bags 130 cm in length), which was used to sample the undergrowth, as well as herbaceous vegetation and shrubby thickets. This method allows for quick and easy collection of representative material [52,53]. It is one of the most widely used methods of thrips collection [26,54]. At all sites, following the literature [52], 4 × 25 sweeps (4 subsamples = 1 sample) were made with an entomological sweeping net along each of the four transects. From each study site, 128 subsamples were taken (4 × 25 sweeps × 8 dates), which constituted 32 samples. Each time, the contents of the scoop were placed in presigned plastic bags. Thrips were then collected under laboratory conditions using a thin brush (size 1) and placed in preservative liquid (70% ethyl alcohol with glycerine in a 9:1 ratio). Further analysis of the collected material required preparation of microscopic slides according to the procedures described by Zawirska [55]. Adult insects were identified to species using the thrips identification keys of Zawirska [55], Schliephake and Klimt [44], and zur Strassen [56]. Based on the literature, the collected species were classified into phagic and zoogeographic groups [44,56].

### 2.3. Data Analysis

The thrips assemblage attributes, such as composition, structure, abundance, dominance, frequency, species richness, diversity, and evenness, were evaluated. Dominance was determined based on the dominance coefficient (D) according to Kasprzak and Niedbała [57]. Based on the value of the dominance coefficient, five classes of dominance were distinguished: eudominants (ED) > 10.00%, dominants (D) (5.01% < D < 10.00%), subdominants (SD) (2.01% < D < 5.00%), recedents (R) (1.01% < D < 2.00%), and subrecedents (SR) (D < 1.00%).

The diversity was calculated by the application of the Shannon index [58], the Gini–Simpson index [58], and evenness by the application of Pielou’s index [58].

The significance of differences between biodiversity index values was tested using the nonparametric Kruskal–Wallis rank ANOVA test. As this test only reports the fact that there are differences between groups but does not report between which pair these differences occur, further post hoc Dunn’s test for multiple comparisons of mean ranks was used. Principal component analysis (PCA) was performed on individual samples from 2014–2015, taking into account species associated with herbaceous plants (excluding arboreal and predators) and occurring in at least 5 samples.

Statistical analyses assumed a significance level of *p* = 0.05. Analyses were performed using IBM SPSS Statistics 23.0. Diversity indices were calculated using Past3.

## 3. Results and Discussion

During the two-year study (2014–2015), 30 species of thrips were collected, which belonged to three families: Aeolothripidae Uzel, 1895 (4 species); Thripidae Stephens, 1829 (24 species); and Phaeolothripidae Uzel, 1895 (2 species): Terebrantia (28 species) and Tubulifera (2 species) (Table 1). Elements widespread in the Holarctic region include twelve species, the Cosmopolitan and Palearctic regions include five species, the Eurasian region includes four species, the European region includes two species, and the Mediterranean and Western Palearctic include one species (Table 1).

In the collected material, as many as 21 species were mesohygrophilous, 7 were xerophilous, and the presence of 1 hygrophilous and 1 shade-loving species was noted (Table 1). As many as 18 species of thrips were common to all three meadow–pasture communities (Table 1). The highest number of species (27) was collected from study site 1, with patches of the fox sedge complex *C. vulpine*. In turn, in study site 2, with the false oat-grass complex *Arrhenatheretum elatioris,* and study site 3, with patches of wild elderberry *S. nigra* and clumps of forest rush *S. sylvaticus,* the same number of species (22) was found (Table 1). The analysis of food preferences of the collected thrips allowed us to distinguish two trophic groups: 26 herbivorous species and 4 zoophagous species, with these last ones from the family Aeolothripidae and the genus *Aeolothrips*: *A. albicinctus*, *A. fasciatus*, *A. intermedius*, and *A. versicolor*, which can also supplement their diet with plant food, e.g., by sucking flower pollen [59]. Among the herbivores, 11 species were associated with monocotyledonous plants, mainly grasses and sedges, 12 chose flowers of dicotyledonous herbaceous plants, and 3 preferred leaves, including 2 dendrophilous species associated with deciduous trees. Nine of the grass-living are oligophagous and two are polyphagous, often found in meadows and pastures, as well as in wild clearings and on grasses growing in forests and on their edges, as well as in mid-field thickets [26,30,41,60]. Some of them are also pests of seed crops of forage grasses and cereals [18,61,62]. Among thrips associated with dicotyledonous herbaceous plants, five species were polyphagous and five species were oligophagous, while both dendrophilous species were polyphagous (Table 1).

Throughout the entire study period (2014–2015), the most abundant species were four grass-living species: *Chirothrips manicatus*, *Aptinothrips rufus*, *Chirothrips hamatus*, and *Anaphothrips obscurus*. The first of those, *Ch. manicatus*, was a definite eudominant at all three sites (Table 2). In both years of the study, two peaks of this species were noted, which occurred at the turn of May and June and in the first days of July. The second most abundant species, *A. rufus*, was eudominant at sites 1 and 3, while at site 2 it was dominant.

At site 3, the dominant species was the hygrophilous species *Ch. hamatus*, while at site 2 it was subdominant and at site 1 it was recedent. *A. obscurus* was subdominant at all three sites (Table 2). *Ch. manicatus* is common in Poland, feeding and reproducing mainly in panicles and ears of grasses, including cereals, as well as other monocotyledonous plants. Mostly females overwinter in wild grasses. It is a dioecious, early-successional species of meadow grass and especially meadow foxtail *A. pratensis* [18,52]. Its abundant occurrence at all three sites was probably related to the presence of fox sedge *C. vulpinae,* especially at site 1, tall ryegrass *A. elatius* and common cocksfoot *D. glomerata* at site 2, and clumps of wood rush *S. sylvaticus* at site 3. Moreover, in all the studied sites, the occurrence of other grasses from the Poaceae family was also noted, on which this species readily feeds. *Ch. manicatus* often has a significant share, especially in groupings inhabiting cereal crops [61] and meadows of different nature [20], and is also found in forest environments [14,63]. Sęczkowska [20,21] reported numerous occurrences of *Ch. manicatus* in meadows near Puławy and in the vicinity of Lublin, while Pobożniak and Grabowska [64] found it in the Nowohuckie Meadows of Kraków. It is a xerophilous species and has been reported in large numbers from xerothermic communities, among others, in the northern part of the Kraków-Częstochowa Upland [29]. Kalinka [65] included it among the characteristic species associated with xerothermic communities with the mosaic of *Festucetum-pallentis*, *Origano-Brachypodietum*, and *Potentillo albae* in the Ojców National Park. Kucharczyk [26] found it to occur in as many as eight plant communities of Roztocze, including the sedge *Caricetum paradoxae* and *Caricetum gracilis* complex, the *Arrhenatheretum elatioris* ryegrass meadow, the *Koelerio glaucae*-*Corynephoretea* thermophilous grass-loving, as well as in the community in the habitat type of fresh pine forest *Leucobryo-Pinetum* and the *Tilio-Carpinetum* oak–hornbeam. Sierka and Sierka [30] found it to be dominant in five of the seven plant communities of the Jaworznickie Hills, with the highest values of dominance in the thermophilous *Trifolio-Agrimonietum* groundcover community and *Pruno-Crategetum* midland scrub.

The second most collected species, *A. rufus*, is also very common throughout Poland, occurring frequently and in large numbers on meadows and pastures and wild grasses in the undergrowth of forests. It prefers mainly grasses from the *Poaceae* family. It also occurs in cereal crops, although less abundantly, and mainly on the edges of fields, to which it migrates from neighboring field margins [61]. It overwinters in clumps of grasses. This species is active from early spring when grass vegetation begins. At first, it feeds and lays eggs in grass leaves, then the larvae feed in leaf sheaths and the females of the next generation lay eggs in the stems, and the larvae that hatch from them feed in panicles and spikes, destroying them [61]. It is xerophilous; Kucharczyk [26] showed it in the plant association of the narrow-leaved elecampane *Inuletum ensifoliae* and the Roztocze forest association of the fertile Carpathian beech *Dentario glandulosae-Fagetum*.

Another dominant species, *Ch. hamatus*, was abundant at sites 2 and 3. It is an oligophage feeding on many grasses, although it particularly prefers meadow foxtail *A. pratensis*, which in turn grew in large numbers, especially at site 2. It is a hygrophilous species. It was noted that this species was more abundant at site 3, especially in areas closer to watercourses where the site was wetter. In addition to the presence of *A. pratensis* at site 2, the increased humidity of this habitat associated with the woodland surrounding it on three sides was probably a factor in its abundance [56,66,67]. This species was found in great numbers in the Nowohuckie Meadows of Kraków, especially in the slightly wet areas of the meadowsweet and marsh cranesbill *Filipendulo-Geranietum* association and the *Cirsietum rivularis* meadow [64].

In contrast, the subdominant *A. obscurus* is a cosmopolitan polyphagous especially associated with vegetations of the *Poaceae* family. It is commonly distributed on meadows and forest grasses and cereals, especially wheat and rye. It is a mesohygrophilous species, although it is also abundant in wetter environments [61]. Sierka and Sierka [30] concluded that it was the dominant species in sweeping net samples from a part of the *Tilio-Carpinetum typicum* oak–hornbeam forest complex. Kucharczyk [26] mentioned it from, among others, the fibrous tussock sedge *Caricetum paradoxae* complex and the tall ryegrass *Arrhenatheretum elatioris* of Roztocze.

The four dominant grass-loving species mentioned above have also been collected by many authors from the undergrowth of beech forests in the Sandomierz Basin [15,41] and the Niski Beskids mountains, as well as in the Sanocko-Turczańskie Mountains [41]. In addition, *Ch. manicatus* was collected in a complex of fertile Carpathian beech forests in the vicinity of Zawoja, and *A. obscurus* in the forests of the Kraków-Częstochowa Jurassic Highland and in the forests of the Roztocze National Park, where *A. rufus* was also observed [41]. These authors also reported that *A. obscurus*, *A. rufus*, *Ch. Manicatus,* and species such as *Aptinothrips stylifer* and *Haplothrips aculeatus* (subrecedents), which we also collected from all sites, were more common in acidic beech and on the edges of fertile beech, and *Ch. hamatus* especially in humid sites and near sources. Of the remaining grass-loving species, *Limothrips denticornis*, which was recedent at sites 2 and 3, was also among the subdominants at site 1 (Table 3). It is an early and widespread species throughout Poland. Its females feed in the spikes and panicles of grasses before grass eating, later entering the leaf sheaths where they lay their eggs. It is found in large numbers on cereals and other grasses, especially *Poaceae* [56,61,68]. It was also abundantly observed in other communities, such as in the undergrowth of *Tilio-Carpinetum stachyetosum* and less numerously in the undergrowth and litter of the *Tilio-Carpinetum typicum* complex of the Bachus Reserve [54] and the *Arrhenatheretum elatioris* complex of the Ojców National Park [65]. This author states that individuals of this species and *Ch. manicatus* are often found on dicotyledonous plants. The possibility of mass occurrence of thrips on species unrelated to their biology is also described by other authors [52]. Also, Pobożniak and Gaborska [64] observed the abundant occurrence of another grass-loving species, *Ch. Manicatus,* in the flowers of red clover *T. pratense* in the Nowohuckie Meadows of Kraków. The remaining grass-loving species *Chirothrips aculeatus*, *Limothrips consimilis*, and *Stenothrips graminum* were counted as subrecedents, and only *Limothrips cerealium* as recedent, at site 1 (Table 2).

The flower-living species, *Haplothrips leucanthemi,* was classified as subdominant at site 1. At the other sites it was recedent (site 3) and subrecedent (site 2). It is a species found in the flowers of *Asteraceae,* with a particular predilection for *Leucanthemum* sp. It is also found in the flowers of *Fabaceae*, especially red clover *T. pratense* and white clover *Trifolium repens* [69].

All the other flower-living species we found were recedents and subrecedents (Table 3). Flower-living species collected from all sites in both years of the study included *Frankliniella intonsa*, *Neohydatothrips gracillicornis* and species of the genus *Thrips*: *T. fuscipennis*, *T. major*, and *T. physapus*. The other species, *T. atratus*, *T. trehernei*, and *T. validus,* were collected in smaller numbers and only from some sites (Table 2). The highest number and share of these species were found at site 1, where the largest number of flowering meadow plants from the *Asteraceae* and *Caryophyllaceae* families were also observed, which are mainly preferred by *T. atratus*, *T. physaphus*, *T. trehernei*, and *T. validus. F. intonsa* was found at all sites, although it was most numerous at site 3. *F. intonsa* is a three-generation, polyphagous species that feeds on flowers and young leaves of dicotyledonous plants [52,70], often found in large numbers in meadow communities [20,21,63] and other communities, including forest ones [26,36,67]. Single individuals of *Melanthrips pallidor*, which prefers flowers of *Brassicaceae* plants, were collected from sites 1 and 3. Associated with flowers of *Fabaceae* were three species: *Odontothrips loti*, *Neohydatothrips abnormis*, and *Neohydatothrips gracillicornis*. While *O. loti* is a species commonly found on wild and cultivated vegetation, on which it even causes economic damage, *N. abnormis* is a less common species [71]. It prefers xerothermic biotopes; in Poland it is known on the Masovian Lowland, the Lublin Upland, and the Sandomierz Lowland [15,72]. In the area we studied, one individual of this species was caught at site 2. In turn, *N. gracillicornis* is found mainly in flowers of plants of the genus *Vicia*. This species occurs at warm locations [73], most often on the edges of forests [74]. Sierka and Sierka [60] included it among the species distinguishing shrubby thickets.

Of the predatory species, only *A. intermedius* and *A. albicinctus* were found at all sites. On the other hand, *A. fasciatus* and *A. versicolor* were present only at site 1. The most abundant was *A. intermedius*, which was recedent, while the others were classified as subrecedents (Table 2). *A. intermedius* occurs in large numbers throughout Poland, mainly on herbaceous and shrubby plants. It sucks up the larvae of other thrips, as well as aphids and the larvae and eggs of other small insects [28]. It can supplement its diet by sucking up the contents of plant tissues, mainly flower petals [75]. Taking in a variety of food, this species grows rapidly and produces large numbers of offspring [59]. Sierka and Sierka [60] showed its presence in all communities of the Jaworznickie Hills, but the highest share was in the assemblage of humid meadows *Molinietum medioeuropaeum* and ryegrass meadow *Arrhenatheretum elatioris*. Also, Kucharczyk [26] showed its presence—among others—in the *Arrhenatheretum elatioris* ryegrass meadow complex, in the *Cairci Agrostidetum-Caninae* acid sedge meadow complex, and in the *Leucobryo-Pinetum* Roztocze pine forest complex. *A. fasciatus* is also associated with flowers of various dicotyledonous plants, while *A. albicinctus* is found in the ground parts of grasses [20,24]. In turn, *A. versicolor* is classified as a dendrophilous species and is found mainly in forests [26,44] and thermophilic thickets [24], where it feeds on flowers and leaves of *Betula* spp., *Corylus* spp., *Quercus* spp., and *Tilia* spp. [23,44,76]. Sierka [77] considered it a characteristic species of oak–hornbeam forests. Kucharczyk and Sęczkowska [54] found the presence of *A. albicinctus*, *A. intermedius,* and *A. versicolor* in the undergrowth of two oak–hornbeam forest subgroups: *Tilio-Carpinetum typicum* and *Tilio-Carpinetum stachyteosum* of the Bachus reserve (Lublin Upland, Chełm Landscape Park), and Kucharczyk [26] noted the occurrence of *A. albicinctus* and *A. versicolor* in oak–hornbeam forests of Roztocze (Tarnawa). All the predator species we found also occurred in the undergrowth of deciduous forests of the central part of the Sandomierz Basin [15].

During the study, two other species associated with deciduous trees were also collected: *Dendrothrips ornatus* (at sites 1 and 2) and *Thrips minutissimus* (at site 1). Both were classified as subrecedents (Table 2). In mass occurrence, feeding larvae and imago of both species can damage the leaf and flower buds of trees and shrubs. *D. ornatus* prefers leaf blades of *Populus alba*, *Salix* spp., *Tilia cordata* [23], and *Ligustrum vulgaris* [78]. The occurrence of this species is generally associated with the vicinity of trees, and it probably arrives in meadows by accident [24,66]. Kucharczyk and Kucharczyk [15,41] showed the presence of *D. ornatus* in the beech forests of Roztocze (Tarnawa) and the oak–hornbeam forests of the central part of the Sandomierz Basin. In turn, *T. minutissimus* was recorded from most of the studied forest communities in Poland and is also frequently found on herbaceous plants of the ground cover [14,15,26,54]. Sierka [77] included it among the dominant species of the oak–hornbeam association *Tilio-Carpinetum typicum*.

The greatest diversity was observed in the thrips fauna of study site 1, which contained meadow species characteristic of the alliance *Arrhenatherion elatioris* and patches of the fox sedge complex *Carex vulpine*. From this study area, 27 thrips species were collected. In turn, from the fresh ryegrass meadow of the alliance *Arrhenatherion elatioris* (site 2), despite a smaller number of species (22), the number of adults collected was slightly higher than those collected from site 1 (Table 3).

Lewis [52] states that the number of thrips species in a given plant community depends mainly on the diversity of the plant community, while the number of individuals of a given species depends on the weather pattern and the presence of other species. Research by many authors indicates that meadow communities, rich in flowering plants, are often rich food reservoirs for these insects. This was pointed out by Kucharczyk [26], who, in her research on the fauna of Roztocze meadows in the communities of narrow-leaved Oman *Inuletum ensifoliae* and tall ryegrass *A. elatioris,* found the occurrence of 30 and 23 thrips species, respectively. Also, Kalinka [65], studying the thrips fauna of the Ojców National Park, observed the highest value of species diversity indices in *Arrhenatheretum elatioris* meadows in the Prądnik Valley.

The highest values of all biodiversity indices were found for site 1. With slightly lower values at site 3, the lowest biodiversity was found at site 2 (Table 3). However, the analysis using the Kruskal–Wallis test for the values of indices calculated for each sample did not show any statistically significant differences in the mean results of biodiversity indices calculated between individual sites for both years together (Table 4).

When analyzing the thrips fauna in the following years, we found that at site 1 the number of species was identical in 2014 and 2015 (23), while the values of biodiversity indices decreased in the second year of the study. It should be noted that site 1 had the highest values of biodiversity indices in 2014 compared to the other sites and years. In 2015, the highest values of biodiversity indices were obtained for site 3. However, the number of species did not change at this site compared to 2014 (18). The largest increase in biodiversity indices compared to 2014 was recorded for site 2. Only at this site did the number of species increase, from 17 to 20 (Table 5). In 2014, statistically significant differences between sites were found for the Gini–Simpson index (*p* = 0.032) and the Pielou evenness index (*p* = 0.005). Further post hoc analysis showed that for the Gini–Simpson index, site 1 was significantly different from site 2 (*p* = 0.026). Also, for the Pielou index, a difference was found between this pair of sites (*p* = 0.004). Site 1 was characterized by the highest species richness and evenness of these species; for site 2, these indicators took the lowest values. No statistically significant differences were found for the other biodiversity indicators (Table 5).

**Figure 2:**
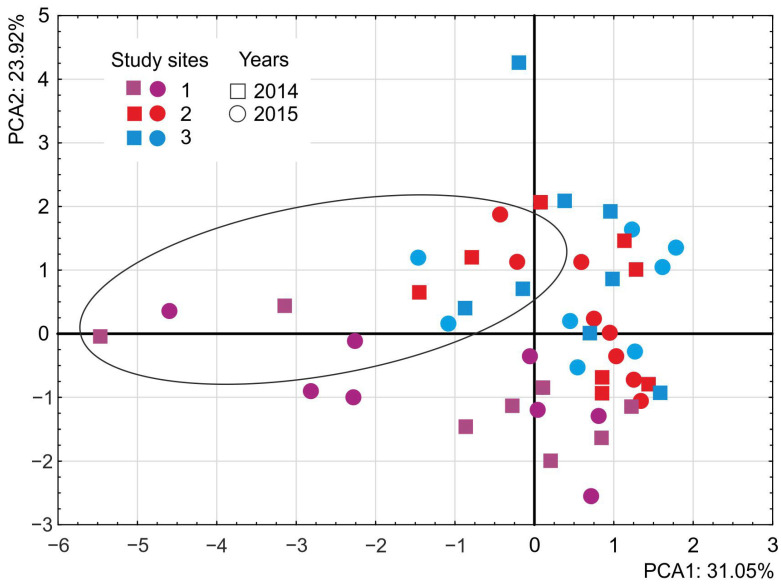
PCA scores plot (1 vs. 2) for a dataset consisting of thrips samples collected at sites 1–3. The ellipse indicates samples collected in May 2014 and 2015.

In 2015, statistically significant differences were found between sites for the number of individuals (*p* = 0.009) and for the Pielou evenness index (*p* = 0.042). Further post hoc analysis showed that for the number of individuals, site 1 was significantly different from site 2 (*p* = 0.017) and site 3 (*p* = 0.036). The highest mean number of individuals was found at site 1; the lowest at the other sites. For the Pielou index, a difference was found between site 1 and site 3 (*p* = 0.036). Site 3 had the highest evenness, while site 1 had the lowest (Table 5). PCA analysis showed differences between species composition in spring (May) samples and summer (June, July, and August). The distribution of samples along the axes separated those taken in spring in both 2014 and 2015 (horizontal axis). These samples mainly contained species wintering at or near the study sites. Of the samples collected in summer, those collected at site 1 form a group that is not significantly mixed with those gathered at sites 2 and 3. This may be due to the use of this area as an occasional pasture with a higher proportion of dicotyledonous herbaceous plants that are hosts for thrips. The other studied areas were under greater anthropogenic pressure, regularly mown (site 2), or used as sustainable pasture for cattle (site 3). Both sites were dominated by grasses or ruderal plants, resulting in a lower diversity of thrips species composition. The samples taken in summer at the last two sites formed a homogeneous group distributed along the vertical axis, except the second sample taken in August 2014 at site 3, which stood out from the others and had the lowest diversity. The graminicolous species *Ch. manicatus*, *A. rufus,* and *A. obscurus* had the greatest impact on the distribution of all summer samples (Figure 2). Very likely, there is also some other hidden factor that distinguishes thrips community in site from the others (site 2 and 3). Analyzing environmental variables and the use of different methods (that indicate these factors) will be beneficial in the future.

In summary, our research indicated that the thrips fauna of the studied sites was quite diverse and rich. We collected 30 species of thrips from the studied meadow–pasture sites of the Góra Bucze, which represents 13.3% of all species recorded from Poland (226) [17], 24% of taxa recorded from the Polish part of the Carpathians (125 species) [32], and 14.2% collected from the Slovak, Polish, Romanian, and Hungarian Carpathians (211 species) [33,34]. The most frequently found species in the Carpathians include *Ch. manicatus*, *A. intermedius*, and *F. intonsa* [33], the first of which was the most abundant from the communities of the Góra Bucze studied by us, while the other two were also present at all sites. All the species we showed, except for *L. cerealium*, were found in different mountain ranges of the Polish Carpathians [32]. *L. cerealium* was present as a recedent at site 1 and 3 and as a subrecedent at site 2 of the Góra Bucze. In turn, its presence in the Stołowe Mountains range of the Sudetes was reported by Stanisławek and Kucharczyk [43]. In turn, the species that was only recorded from the Beskid Mały mountain was *L. consimilis* [32]. In our study, we collected only two individuals of this species from site 3. Both of them are thermophilic species associated with grasses [29]. Initially, it was thought to be a very abundant species of northern and eastern Germany, but very rare in northern and eastern Europe [79]. However, later studies have shown that *L. cerealium* inhabits cereals in Wielkopolska and the Lublin region [19,80], in the West Pomeranian and Pomeranian voivodeships [61], and in southwestern and south-central Poland [81]. Fertilized females of *L. cerealium* overwinter under the bark of trees, in tufts of dry grasses, in empty plant stems, and between the needles, especially of the common juniper *Juniperus communis*. It appears in meadows in May, from where it migrates to cereals. Single individuals of this species have been recorded on a xerothermic grassland that is a mosaic of *Stipetum capillatae* and *Thalictro-Salvietum pratensis* [29]. All developmental stages of this species occur in leaf sheaths and on leaves of wild grasses, including cereals, mainly wheat and oats, and, as the plants grow, in their ears [82]. Its presence at the Góra Bucze sites could have resulted from the fact that there were fields below where cereals were grown. Also noteworthy is the species *N. abnormis* recorded by us from site 3, which had previously been recorded in the Carpathians only from the Babia Góra massif [32].

All the listed dominant grass-living thrips species were recorded from the Carpathians. *Ch. manicatus* was recorded from the Mały Beskid, the Babia Góra massif, the Tatras, the Beskid Sądecki and Beskid Niski, the Bieszczady Mountains, and the Sanocko-Turczańskie Mountains. *A. rufus* and *A. obscurus* were similar, except that *A. rufus* was not recorded in the Beskid Mały mountains, and *A. obscurus* in the Beskid Sądecki Mountains. In turn, *Ch. hamatus* was recorded from the Mały Beskid, the Babia Góra massif, the Niski Beskids, the Bieszczady Mountains, and the Sanocko-Turczańskie Mountains [28,32]. *Ch. manicatus*, *A. rufus*, and *A. obscurus* were also recorded from the central and eastern Sudetes [32].

All other species we found were also reported from different mountain ranges of the Carpathians, and some of them also from the Sudetes: *A. intermedius*, *A. albicinctus*, *A. fasciatus*, *F. intonsa*, *H. aculeatus*, *N. abnormis*, *O. loti*, *T. atratus*, *T. fuscipennis. T. major*, *T. physahus*, *T. tabaci,* and *T. validus* [32,33,43].

## Figures and Tables

**Table 1:** List of thrips (Thysanoptera) species collected in 2014–15 from Góra Bucze landscape–nature complex together with their characteristics.

Species	Study Site			Characteristics
	I	II	III	
Suborder Terebrantia				
*Aeolothrips albicinctus* Haliday, 1836	+	+	+	gr, h, z, sk, po, HOL, *Calamagrostis* sp.
*Aeolothrips fasciatus* Linnaeus, 1758	+	−	−	fl, h, z, me, po, COS
*Aeolothrips intermedius* Bagnall, 1934	+	+	+	fl, h, z, me, po, PAL
*Aeolothrips versicolor* Uzel, 1895	+	−	−	ar, fo, z, me, ol, HOL, *Quercus* sp., *Tilia* sp.
*Anaphothrips obscurus* (Müller, 1776)	+	+	+	gr, h, me, po, COS, *Poaceae*
*Aptinothrips rufus* (Haliday, 1836)	+	+	+	gr, xt, ol, COS, *Poaceae*
*Aptinothrips stylifer* Trybom, 1894	+	+	+	gr, me, ol, COS, *Poaceae*
*Chirothrips aculeatus* Bagnall, 1927	+	−	+	gr, me, ol, EUS, *Poaceae*, *Bromus* sp.
*Chirothrips hamatus* Trybom, 1895	+	+	+	gr, hg, ol, HOL, *Alopecurus pratensis*
*Chirothrips manicatus* Haliday, 1836	+	+	+	gr, xt, ol, HOL, *Poaceae*
*Dendrothrips ornatus* (Jablonowski, 1894)	+	+	−	ar, fo, me, po, EUR, *Syringa* sp., *Ligustrum* sp., *Fraxinus* sp., *Tilia* sp.
*Frankliniella intonsa* (Trybom, 1895)	+	+	+	fl, h, me, po, HOL
*Limothrips cerealium* Priesner, 1926	+	+	+	gr, xt, ol, EUS, *Poaceae*
*Limothrips consimilis* Priesner, 1926	−	−	+	gr, xt, ol, EUS, *Poaceae*, *Bromus* sp.
*Limothrips denticornis* Haliday, 1836	+	+	+	gr, me, ol, HOL, *Poaceae*
*Melanthrips pallidior* Priesner, 1919	+	−	+	fl, xt, po, SBM, *Asteraceae*, *Brassicaceae*, *Fabaceae*
*Neohydatothrips abnormis* (Karny, 1910)	−	+	−	fl, h, xt, ol, EUR, *Fabaceae*, *Astragalus* sp.
*Neohydatothrips gracillicornis* (Williams, 1916)	+	+	+	fl, h, xt, po, PAL, *Fabaceae*, *Vicia* sp.
*Odontothrips loti* (Haliday, 1852)	+	−	−	fl, h, me, ol, HOL, *Fabaceae*, *Lotus* sp.
*Stenothrips graminum* Uzel, 1895	−	+	+	gr, me, po, W-Pal, *Poaceae*, *Avena sativa*, *Triticum* sp.
*Thrips atratus* (Haliday, 1836)	+	−	−	fl, h, me, po, HOL, *Caryophyllaceae*, *Lamiaceae*
*Thrips fuscipennis* (Haliday, 1836)	+	+	+	fl, h, me, po, HOL, *Rosaceae*
*Thrips major* Uzel, 1895	+	+	+	fl, h, me, po, PAL
*Thrips minutissimus* Linnaeus, 1761	+	−	−	ar, fo, me, po, EUS, *Rosaceae*, *Carpinus* sp., *Quercus* sp.
*Thrips physapus* Linnaeus, 1758	+	+	+	fl, h, me, po, PAL, *Asteraceae*
*Thrips tabaci* Lindeman, 1888	+	+	+	fl, fo, h, me, po, COS
*Thrips trehernei* Preisner, 1927	+	+	−	fl, h, me, ol, HOL, *Asteraceae*
*Thrips validus* Uzel, 1895	+	+	+	fl, h, me, ol, HOL, *Asteraceae*
Suborder Tubulifera				
*Haplothrips aculeatus* (Fabricius, 1803)	+	+	+	gr, me, po, PAL, *Poaceae*
*Haplothrips leucanthemi* (Schrank, 1781)	+	+	+	fl, h, me, ol, HOL, *Chrysanthemum* sp.

Note: ar—arboricolous, fl—floricolous, fo—follicolous, gr—graminicolous, h—herbicolous, z—zoophagous, hg—hygrophilous, me—mesohygrophilous, xt—xerophilous sk—skiophilous, po—polyphagous, ol—oligophagous, COS—Cosmopolitan, EUR—European, EUS—Eurosiberian, HOL—Holarctic, PAL—Palearctic, W-Pal—West Palearctic, SBM—Sub-Mediterranean (South and Central Europe).

**Table 2:** Dominance of thrips species (Thysanoptera), Góra Bucze, 2014–2015.

Species	Study Site		
	1	2	3
	Share [%]		
*Chirothrips manicatus*	60.31 ED	76.42 ED	65.74 ED
*Aptinothrips rufus*	12.09 ED	5.95 D	13.64 ED
*Chirothrips hamatus*	1.20 R	4.99 SD	5.58 D
*Anaphothrips obscurus*	3.66 SD	4.04 SD	4.49 SD
*Haplothrips leucanthenii*	10.26 ED	0.14 SR	1.14 R
*Limothrips denticornis*	0.37 SR	2.24 SD	3.43 SD
*Limothrips cerealium*	1.27 R	0.77 SR	1.19 R
*Frankiniella intonsa*	0.98 SR	0.68 SR	1.23 R
*Thrips physapus*	1.17 R	0.91 SR	0.15 SR
*Aeolothrips intermedius*	1.27 R	0.59 SR	0.26 SR
*Thrips tabaci*	1.55 R	0.05 SR	0.56 SR
*Haplothrips aculeatus*	0.89 SR	0.94 SR	0.20 SR
*Thrips fuscipennis*	1.12 R	0.41 SR	0.51 SR
*Thrips major*	0.80 SR	0.68 SR	0.56 SR
*Chirothrips aculcatus*	0.66 SR	-	0.51 SR
*Thrips minutissimus*	0.33 SR	-	-
*Aeolothrips albicinctus*	0.47 SR	0.05 SR	0.05 SR
*Dendrothrips ornatus*	0.28 SR	0.18 SR	-
*Neohydatothrips gracillicornis*	0.56 SR	0.05 SR	0.05 SR
*Aeolothrips versicolor*	0.19 SR	-	-
*Aptinothrips stylifer*	0.14 SR	0.36 SR	0.05 SR
*Melanthrips pallidior*	0.05 SR	-	0.31 SR
*Thrips validus*	0.14 SR	0.18 SR	0.15 SR
*Stenothrips graminum*	-	0.14 SR	0.10 SR
*Thrips trehernei*	0.05 SR	0.18 SR	-
*Limothrips consimilis*	-	-	0.10 SR
*Odontothrips loti*	0.09 SR	-	-
*Aeolothrips fasciatus*	0.05 SR	-	-
*Thrips atratus*	0.05 SR	-	-
*Neohydatothrips abnormis*	-	0.05 SR	-

Kasprzak and Niedbała (1981): eudominant (ED) > 10.00%, dominant (D) (5.01% < D < 10.00%), subdominant (SD) (2.01% < D < 5.00%), recedent (R) (1.01% < D < 2.0%), subrecedents (SR) (D < 1.00%).

**Table 3:** Biodiversity indicators, Góra Bucze 2014–2015.

Diversity	Study Site		
	1	2	3
Number of species	27	22	22
Number of specimens	2134	2201	1958
Gini–Simpson index	0.61	0.41	0.54
Shannon index	1.57	1.07	1.32
Pielou index	0.48	0.35	0.43

**Table 4:** Kruskal–Wallis one-way analysis of variance by ranks of biodiversity indicators, comparison of study sites, all research period (2014–2015), Góra Bucze.

Index	Study Site			Kruskal–Wallis Test	
	1	2	3		
	Mean (±SD)	Mean (±SD)	Mean (±SD)	*χ* ^2^	*p*
Number of species	5.17 ± 2.81	4.14 ± 2.47	4.30 ± 2.73	4.80	0.091
Number of specimens	33.34 ±22.70	34.39 ± 30.80	30.59 ± 23.17	1.00	0.606
Gini–Simpson index	0.46 ± 0.26	0.37 ± 0.25	0.44 ± 0.25	4.40	0.111
Shannon index	0.96 ± 0.59	0.76 ± 0.60	0.88 ± 0.53	3.72	0.156
Pielou index	0.65 ± 0.22	0.60 ± 0.21	0.67 ± 0.19	3.30	0.192

*p* ≤ 0.05 = significant differences. For statistical calculations, an index calculated for each sample was used.

**Table 5:** Kruskal–Wallis one-way analysis of variance by ranks of biodiversity indicators, comparison of study sites in 2014 and 2015, Góra Bucze.

Index	Study Site			Kruskal–Wallis Test	
	1	2	3		
	Mean (±SD)	Mean (±SD)	Mean (±SD)	*χ^2^*	*p*
	Year 2014				
Number of species	5.09 ± 2.93 a *	4.22 ± 2.54 a	4.53 ± 3.01 a	1.61	0.448
Number of specimens	29.59 ± 21.35 a	46.66 ± 36.64 a	38.47 ± 27.53 a	2.65	0.266
Gini–Simpson index	0.49 ± 0.27 a	0.32 ± 0.22 b	0.41 ± 0.26 ab	6.90	0.032
Shannon index	1.04 ± 0.64 a	0.66 ± 0.45 a	0.81 ± 0.54 a	5.75	0.056
Pielou index	0.72 ± 0.17 a	0.53 ± 0.21 b	0.61 ± 0.22 ab	10.46	0.005
	Year 2015				
Number of species	5.25 ± 2.72 a	4.06 ± 2.45 a	4.06 ± 2.46 a	4.52	0.105
Number of specimens	37.09 ± 23.72 a	22.13 ± 16.59 b	22.72 ± 14.31 b	9.33	0.009
Gini–Simpson index	0.43 ± 0.25 a	0.43 ± 0.26 a	0.48 ± 0.24 a	0.90	0.637
Shannon index	0.88 ± 0.53 a	0.86 ± 0.55 a	0.96 ± 0.51 a	0.68	0.711
Pielou index	0.58 ± 0.24 b	0.67 ± 0.19 ab	0.73 ± 0.14 a	6.36	0.042

* Means within a row followed by the same letter(s) do not differ significantly (Dunn’s test; *p* < 0.05). For statistical calculations, the index calculated for each sample was used.

## Data Availability

The data presented in this study are available on request from the corresponding author.
